# Supplementary material for: Developmental dopamine loss rewires striatal circuits to promote locomotion
Source: Mol Neurodegener. 2026 Jan 3;21:7. doi: 10.1186/s13024-025-00920-2 (PMC12866494; doi:10.1186/s13024-025-00920-2)
Supplement: Supplementary file 1 — Supplementary Material 1 [file 13024_2025_920_MOESM1_ESM.pdf]

Title: Developmental Dopamine Loss Rewires Striatal Circuits to Promote Locomotion

Authors: Jie Dong[1,2], Breanna T. Sullivan[1], Victor M. Martinez Smith[1], Lupeng Wang[1], Lulu Tian[1,3], Justin Kung[1], Bin Song[1, 4, 5], Shirong Lin[1, 6, 7], Andreanna Le[1], Lixin Sun[1], Lisa Chang[1], Jinhui Ding[8], Weidong Le[9, *], Jun Jia[1, 10], and Huaibin Cai[1, *]

Supplementary Figures and Figure Legends

Fig. S1. Loss of PITX3 expression in the midbrain DANs of *Pitx3*$^{ak/ak}$ mice

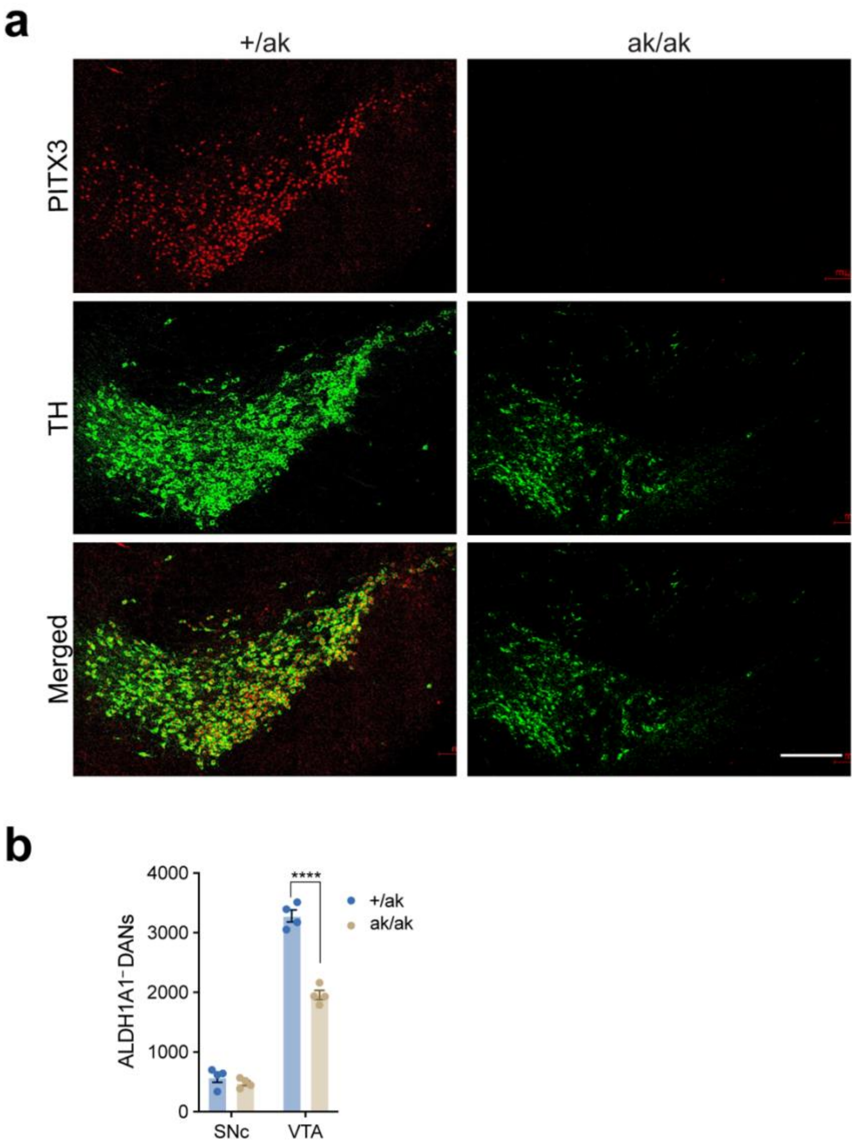

a Representative image showing PITX3 (red) and TH (green) immunolabeling in the midbrain of *Pitx3*$^{+/ak}$ and *Pitx3*$^{ak/ak}$ mice. Scale bar, 200 µm.

b Percentage of ALDH1A1-negative (ALDH1A1^{-}) cells among TH^{+} cells in the SNc and VTA. Two-way ANOVA followed by Sidak's post hoc correction: Genotype, F (1, 6) = 109.4, $^{****}p <$ 0.0001; Region, F (1, 6) = 565.7, $^{****}p < 0.0001$; Interaction, F (1, 6) = 49.05, $^{***}p = 0.0004$. Multiple comparisons: SNc, $p = 0.6433$; VTA, $^{****}p < 0.0001$. N = 4 mice per group

Fig. S2. No significant difference in the number of interneurons between *Pitx3*$^{+/ak}$ and *Pitx3*$^{ak/ak}$ mice

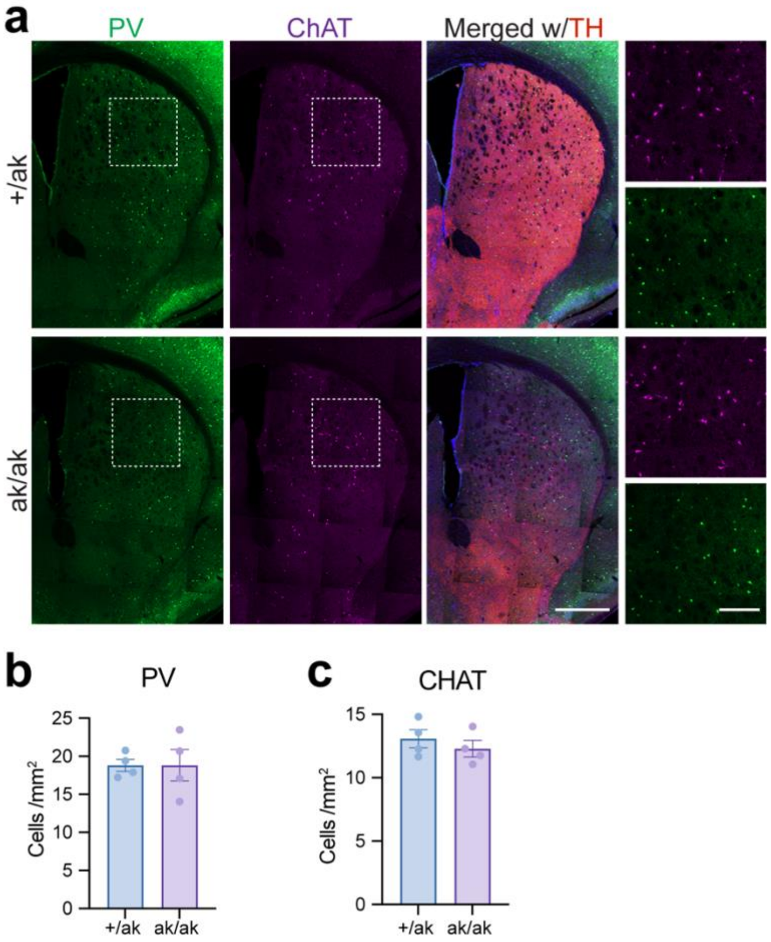

a Representative coronal images of the dorsal striatum stained for PV (green), ChAT (magenta) and TH (red) in *Pitx3*$^{+/ak}$ and *Pitx3*$^{ak/ak}$ mice. Right panels show magnified views of the dotted squares in the left panels. Scale bar: 500 µm (left) and 200 µm (right).

b Comparison of the PV^{+} cell density in the dorsal striatum between *Pitx3*$^{+/ak}$ and *Pitx3*$^{ak/ak}$ mice. Unpaired t test, n = 4 mice per each genotype, t (6) = 0.0055, *p* = 0.996.

c Comparison of the ChAT^{+} cell density in the dorsal striatum between *Pitx3*$^{+/ak}$ and *Pitx3*$^{ak/ak}$ mice. Unpaired t test, n = 4 mice per each genotype, t (6) = 0.8309, *p* = 0.438.

Fig. S3. No apparent change of dSPN projections to the GPe of *Pitx3*$^{ak/ak}$ mice.

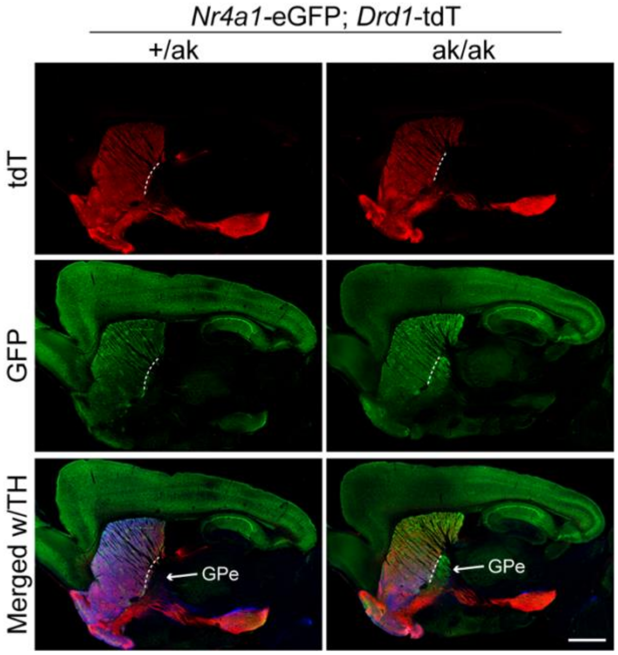

Representative sagittal sections of *Nr4a1*-eGFP; *Drd1*-tdT mice on *Pitx3*$^{+/ak}$ and *Pitx3*$^{ak/ak}$ backgrounds. Scale bar: 1000 μm
